# HpSlyD inducing CDX2 and VIL1 expression mediated through TCTP protein may contribute to intestinal metaplasia in the stomach

**DOI:** 10.1038/s41598-017-02642-y

**Published:** 2017-05-23

**Authors:** Qiuping Li, Yanmei Zhu, Jun Liu, Xiuwen Yu, Moye Chen, Nannan Dong, Yuehua Gong, Yuan Yuan

**Affiliations:** 1 Tumor Etiology and Screening Department of Cancer Institute and General Surgery, the First Affiliated Hospital of China Medical University, and Key Laboratory of Cancer Etiology and Prevention (China Medical University), Liaoning Provincial Education Department, Shenyang, 110001 China; 2 Mary Babb Randolph Cancer Center, West Virginia University, Morgantown, WV 26506 USA; 3 Department of Physiology and Pharmacology, West Virginia University, Morgantown, WV 26506-9229 USA; 4 Department of Pathology, Cancer Hospital of China Medical University; Liaoning Cancer Hospital & Institute, Shenyang, 110042 Liaoning Province China; 5 Department of Pathology, Qiqihar Medical College, Qiqihar, Heilongjiang China

## Abstract

*Helicobacter pylori* infection is the most important risk factor for gastric intestinal metaplasia (IM). Our previous study demonstrated that infection with *H. pylori HpslyD*-positive strains associated with IM. To further investigate the signalling pathway involved in HpSlyD-induced IM, CDX2 and VIL1 expressions were determined before and after HpSlyD application. TCTP was knocked down by siRNA or overexpressed by plasmid transfection. An HpSlyD binding protein was used to block HpSlyD’s enzymatic activity. The expression of CDX2 and TCTP in gastric diseases was measured by immunohistochemistry. Our results showed HpSlyD induced CDX2 and VIL1 expressions. TCTP protein expression was markedly increased after application of HpSlyD and in an HpSlyD-expressing stable cell line. Downregulation of TCTP protein led to decreased HpSlyD-induced CDX2 and VIL1. Overexpression of TCTP protein improved the expression of CDX2 and VIL1. Co-application of HpSlyD and FK506 led to significant reductions in CDX2, VIL1, and TCTP expression. Immunohistochemistry demonstrated that CDX2 and TCTP expression was higher in *HpslyD*-positive specimens compared with *HpslyD*-negative ones. Expression of CDX2 was positively correlated with TCTP in *HpslyD*-positive cells. Our study is the first to show that HpSlyD induction of CDX2 and VIL1 expression mediated through TCTP may contribute to IM in the stomach.

## Introduction

Gastric intestinal metaplasia (GIM) is a phenomenon of gastric mucosa morphological and functional differentiation into an intestinal-type phenotype, and it is a manifestation of gastric mucosa deviating from its normal phenotype during tissue repair. GIM is the main histopathological change associated with atrophic gastritis. As a repair process occurring after injury that leads to pre-cancer, GIM is closely related to the development of intestinal-type gastric cancer, and its formation can increase the risk of gastric cancer more than 10 times^1^. Understanding the occurrence, persistence and development of GIM is very important for the prevention and treatment of intestinal-type gastric cancer.

Studies have shown that multiple risk factors are associated with the occurrence of GIM, including *Helicobacter pylori* infection, a high salt diet, consuming a lot of smoked or canned food, smoking, alcohol consumption and chronic bile reflux. Among these, *H. pylori* infection is the most important risk factor for GIM, and it can increase the risk of GIM 4.5 to 9 times^2^. Correa believes that *H. pylori* infection causes a series of pathological changes in the gastric mucosa, including superficial gastritis, atrophic gastritis, atypical hyperplasia and gastric carcinogenesis^3^. Although the causal relationship between *H. pylori* infection and GIM is an indisputable fact, GIM only develops in 30% of patients with *H*. *pylori* infection, and only 7% of these individuals go on to develop intestinal-type gastric cancer^4^. Studies have shown that different strains of *H. pylori* carrying different virulence factors are associated with different histopathological changes of the gastric mucosa. For example, the strain carrying *cagA* and *vacA* can produce a stronger inflammatory response, which is related to the occurrence of precancerous lesions such as GIM^5^. In a previous study, we identified a novel peptidylproline cis-trans-isomerase (PPIases, EC number 5.2.1.8) associated with gastric carcinogenesis, which encodes the protein *H. pylori* SlyD (HpSlyD)^6^. HpSlyD has the ability to promote cell proliferation, malignant transformation and invasion, and to inhibit apoptosis^7, 8^. Further study has shown that infection with *HpslyD*-positive strains may be associated with atrophic gastritis^9^. However, the signalling pathway involved in HpSlyD-induced intestinal metaplasia is not yet completely understood.

Caudal-related homeobox 2 (CDX2) is a molecular engine that regulates intestinal differentiation. It can directly promote the expression of a variety of intestinal cell-specific factors, while playing an irreplaceable role in maintaining intestinal cell proliferation, development and differentiation. Under normal conditions, CDX2 expression is restricted to the intestine, but it is ectopically expressed in IM lesions, not only of the stomach, but also of the oesophagus and gall bladder, among other locations. CDX2 activation plays a key role in the development of GIM^10^. Villin 1 (VIL1) is a structural protein involved in the formation of small intestinal microvilli and has upregulation of expression in IM. VIL1 is a known transcriptional target of CDX2^11^. Both CDX2 and VIL1 play a key role in the development of gastric metaplasia. It has been reported in the literature that *H. pylori* can affect CDX2 and VIL1 expression^12–14^. However, it is unclear whether HpSlyD affects CDX2 and VIL1 expression, and if it does, how it regulates CDX2 and VIL1 transcriptional expression is also unclear.

Translationally controlled tumor protein (TCTP), a highly conserved protein found in eukaryotic cells, is an important tumor-associated protein identified in a study of tumor reverse screening. In 2007, the journal Nature reported^15^ that TCTP controls growth and differentiation in drosophila and TCTP overexpression occurs in many human cancers, such as breast cancer and liver cancer^16–21^. Recent studies have shown that TCTP is also pivotal in the cell reprogramming network, with a role as a checkpoint, and it regulates the transition points of cell phenotype under a variety of physiological and pathological states^22^. It is unclear whether TCTP is involved in the regulation of GIM. In our previous study, using differential proteomics, we screened for changes in protein expression associated with the expression of HpSlyD in a stable cell line. Among the 21 up-regulated proteins, the one elevated the most was TCTP, suggesting that TCTP may be involved in HpSlyD-mediated regulation (data not shown). However, this speculation needs to be further verified.

In this study, we investigated whether HpSlyD could induce CDX2 and VIL1 expression *in vivo* and *in vitro* and whether TCTP regulates CDX2 and VIL1 expression induced by HpSlyD, and we aimed to clarify the signalling pathway involved in HpSlyD-induced IM in the stomach.

## Materials and Methods

### Cell culture and treatment

The human gastric carcinoma cell lines AGS and N87 were purchased from the American Type Culture Collection (ATCC, Manassas, VA, USA). They were grown in Ham’s F-12 medium (HyClone, USA) or Dulbecco’s modified Eagle’s medium (DMEM; HyClone, USA) supplemented with 10% foetal bovine serum (FBS, Gibco, Australia) in an atmosphere consisting of 5% CO_2_ at 37 °C. AGS cells were transfected with either *SlyD-GFP* or *GFP* plasmids and stable cell lines were obtained using the methods described by Zhu *et al*.^8^. N-terminal His tagged SlyD was purified by Ni^2+^ affinity chromatography as described earlier^7^. For all experiments, HpSlyD was used at a concentration of 200 ng/mL. The HpSlyD binding protein tacrolimus (FK506) was purchased from Astellas Ireland Co., Ltd., dissolved in ddH_2_O at a concentration of 18 mg/ml and stored at −20 °C until use.

### RNA extraction and Real-time quantitative RT-PCR (qPCR)

Total RNA was extracted using TRI Reagent (Ambion, USA) and converted to cDNA using a PrimeScript RT reagent kit (Takara, Japan). Human CDX2 (forward 5′-TTCACTACAGTCGCTACATCACC-3′; reverse 5′-TTGTTGATTTTCCTCTCCTTTGC-3′) and VIL1 (forward 5′-GGCAAGAGGAACGTGGTAGC-3′; reverse 5′-CGGTCCATTCCACTGGATGA-3′) were amplified with SYBR Green (SYBR Premix Ex Taq II, Takara, USA) in a fluorescence reader ABI Prism 7500. The following PCR parameters were used: 95 °C for 30 seconds, 40 cycles of 95 °C for 15 seconds, 55 °C for 30 seconds and finally an elongation step at 72 °C for 30 seconds. Each reaction was performed in triplicate and normalized to *GAPDH*. Relative expression of the target genes was determined using the 2^−ΔΔCt^ method^23^. Thereafter, expression was expressed as fold difference relative to that of the untreated control cells. The results are expressed as mean ± SD of representative triplicates.

### Protein extraction and western blot

Western blot analysis was performed using standard techniques. Briefly, cells (2 × 10^6^/well) were treated with or without SlyD (200 ng/mL) for 40 hours. Total protein was extracted using a lysis buffer (2% mercaptoethanol, 20% glycerol, and 4% SDS, in 100 mM Tris–HCl buffer, pH 6.8). Equal amounts of total protein (60 µg/lane) were separated and transferred to PVDF membranes (Bio-Rad, Hercules, CA). The membranes were incubated with primary antibodies overnight at 4 °C: rabbit monoclonal anti-CDX2 (1:2000, Abcam, USA), mouse monoclonal anti-VIL1 (1:2000, Origene, USA), rabbit monoclonal anti-TCTP (1:250, Abcam, USA) and then with the appropriate horseradish peroxidase-conjugated secondary antibody (Zhongshan Golden Bridge Biotechnology Co. Ltd, Beijing, China) and visualized by enhanced chemiluminescence (Solarbio, China).

### TCTP RNA interference and overexpression

A small interfering RNA (siRNA) duplex targeting TCTP (5′-GAAATCAATCAAAGGGAAA-3′) and a nonsilencing control siRNA duplex were synthesized by RIBOBIO (Guangzhou, China). A TCTP expression plasmid and a control plasmid were purchased from Origene (Beijing, China). Cells were cultured in antibiotic-free medium for 2 hours. They were then transfected with TCTP siRNA (50 nM) or TCTP plasmid DNA (2.5 ng/ul) using Lipofectamine 2000 (Invitrogen, USA). Silencing was evaluated 40 hours after transfection by western blot. TCTP overexpression was evaluated 24 hours after transfection by western blot.

### PPIase activity assay

SlyD (200 ng/ml) activity was measured in a coupled assay with chymotrypsin^24^ with or without FK506 (18 mg/ml, Astellas Ireland Co., Ltd., Ireland). Cell lysates were incubated with 75 N-succinyl-Ala-Ala-Pro-Phep-nitroanilide (Sigma–Aldrich, St. Louis, MO, USA) in 50 mM Hepes, 100 mM sodium chloride buffer (pH 8.0). The reaction was initiated by adding 16 μm of α-chymotrypsin. The release of p-nitroanilide was monitored spectrophotometrically (Beckman DU-640, Beckman, USA) at 25 °C for 5 minutes by recording the increase in A390.

### Human tissue specimens and immunohistochemistry

Tissue samples were obtained from 84 individuals with gastritis (GS), 91 individuals with intestinal type atrophic gastritis (IM-GA) and 58 with gastric cancer (GC) who participated in the Zhuanghe Gastric Diseases Screening Program between 2008 and 2011, including 133 men and 100 women, 149 cases ≤ 60 years of age and 84 cases > 60 years of age. All subjects were histologically diagnosed based on the updated Sydney System for gastritis. This study was approved by the Ethics Committee of the First Affiliated Hospital of China Medical University Shenyang, China. Written informed consent was obtained from the participants.

All experiments were performed in accordance with relevant guidelines and regulations of the First Affiliated Hospital of China Medical University Shenyang, China. Formalin-fixed, paraffin-embedded tissues were immunohistochemically (IHC) stained using the avidin-biotin complex method as previously described^25^. Mouse anti-human CDX2 monoclonal antibody (Fuzhou Maixin Biotech. Co., Ltd. Fujian, China) and rabbit anti-human TCTP monoclonal antibody (1:250, Abcam, USA) were used.

The IHC results were evaluated and scored independently by two investigators who were blinded to the patients’ clinicopathological characteristics. Protein expression was evaluated using a semi-quantitative scoring criterion based on the staining intensity (0, no staining; 1, light brown staining; 2, brown staining; and 3, heavy brown staining) and proportion of stained epithelial cells (0, ≤ 5%; 1, 5–25%; 2, 25–50%; 3, 50–75%; and 4, ≥ 75%). Staining intensity was measured at the sites of IM glands. The staining intensity was then multiplied to generate an immunoreactivity score (IS) for each specimen^26^.

### DNA extraction and *H. pylori* testing

DNA samples were extracted from the 233 paraffin fixed gastric specimens using a WaxFree^TM^ DNA Kit (Quick DNA preparation for FFEP; TrimGen Corp., USA). *H. pylori* 16s rRNA, *glmM* (formally *ureC*) and *slyD* genes were detected using a PCR method as previously described^27–29^. The primer sequences were as follows: 16s rRNA, forward primer: 5′-CGTTAGCTGCATTACTGGAGA-3′, reverse primer: 5′-GAGCGCGTAGGCGGGATAGTC-3′; *glmM*, forward primer: 5′-AAGCTTTTAGGGGTGTTAGGGGTTT-3′, reverse primer: 5′-AAGCTTACTTTCTAACACTAACGC-3′; *slyD*, forward primer: 5′-CCCACCTTTCTTTCCG-3′, reverse primer: 5′-CCATTCAAGCCACTATCAA-3′. The expected amplification products were 295 bp, 294 bp and 203 bp, respectively. For 16s rRNA and *glmM*, PCR cycling conditions consisted of 35 cycles: 94 °C for 45 sec, 55 °C for 45 sec and 72 °C for 45 sec; for *slyD*, 30 cycles: 94 °C for 45 sec, 57 °C for 45 sec and 72 °C for 45 sec. PCR products were then separated by electrophoresis on a 2% agarose gel. Baseline *H. pylori* infection status was determined based on Hp 16s rRNA and *glmM* PCR amplication. If both two tests were positive, the patient was judged to be *H. pylori* infected.

### Statistical analysis

All analyses were carried out by using SPSS for Windows version 16.0. Data were presented as mean ± SD. Differences in the mRNA and protein expression levels of CDX2, VIL1 and TCTP between the treated and non-treated group were analysed by Student’s t-test. The correlations between *H. pylori* infection in tissue samples with other factors were determined using the bilateral *χ* ^2^ test. Non-parametric tests were used to analyse the differences of CDX2 and TCTP protein detected by IHC. Correlation analysis was performed between TCTP and CDX2 expression. A value of P < 0.05 was defined as statistically significant.

## Results

### HpSlyD induces CDX2 and VIL1 expression in gastric epithelial cell lines

**Figure 1:**
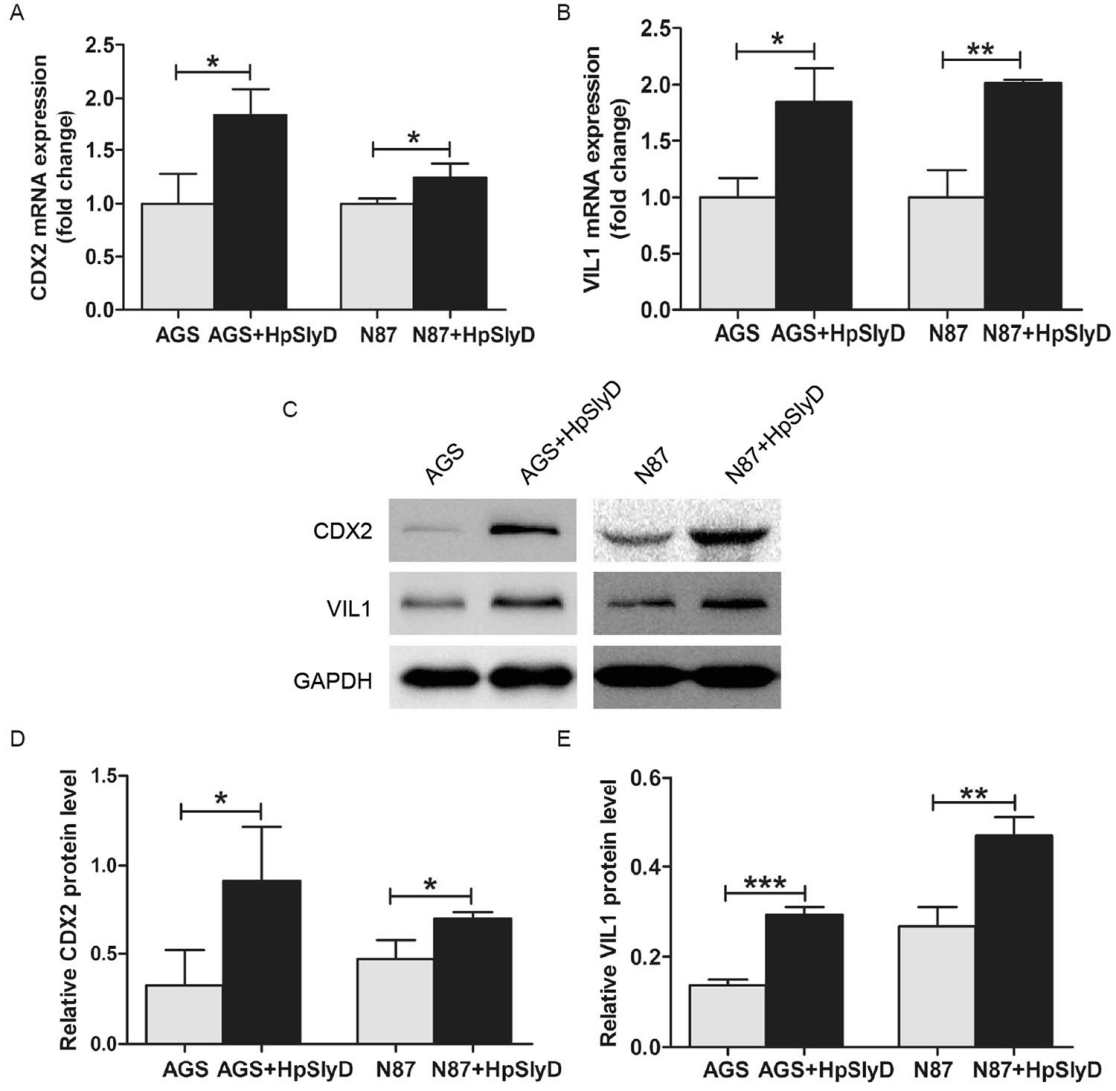
Effect of HpSlyD treatment on CDX2 and VIL1 expressions in gastric epithelial cells. (**A**,**B**) mRNA expression levels of *CDX2* and *VIL1* in AGS and N87 cells treated with 200 µg/ml HpSlyD for 40 hours. The mRNA levels are normalized to *GAPDH* mRNA. The values obtained with non-treated cells are referred to as 1. Results (mean ± SD) from three independent experiments are presented as fold induction. (**C,D,E**) Western blots for CDX2 and VIL1 expression in AGS (**C,D**) or N87 (**C,E**) cells treated with 200 µg/ml HpSlyD for 40 hours. Non-treated cells were used as controls and GAPDH was used as a loading control. The results shown in (**A,B,D**, and **E**) are means ± SD, each experiment performed in triplicate. *P < 0.05, **P < 0.01, ***P < 0.001 as compared with non-treated cells. Full-length gels are presented in Supplemental Figure 1.

**Figure 2:**
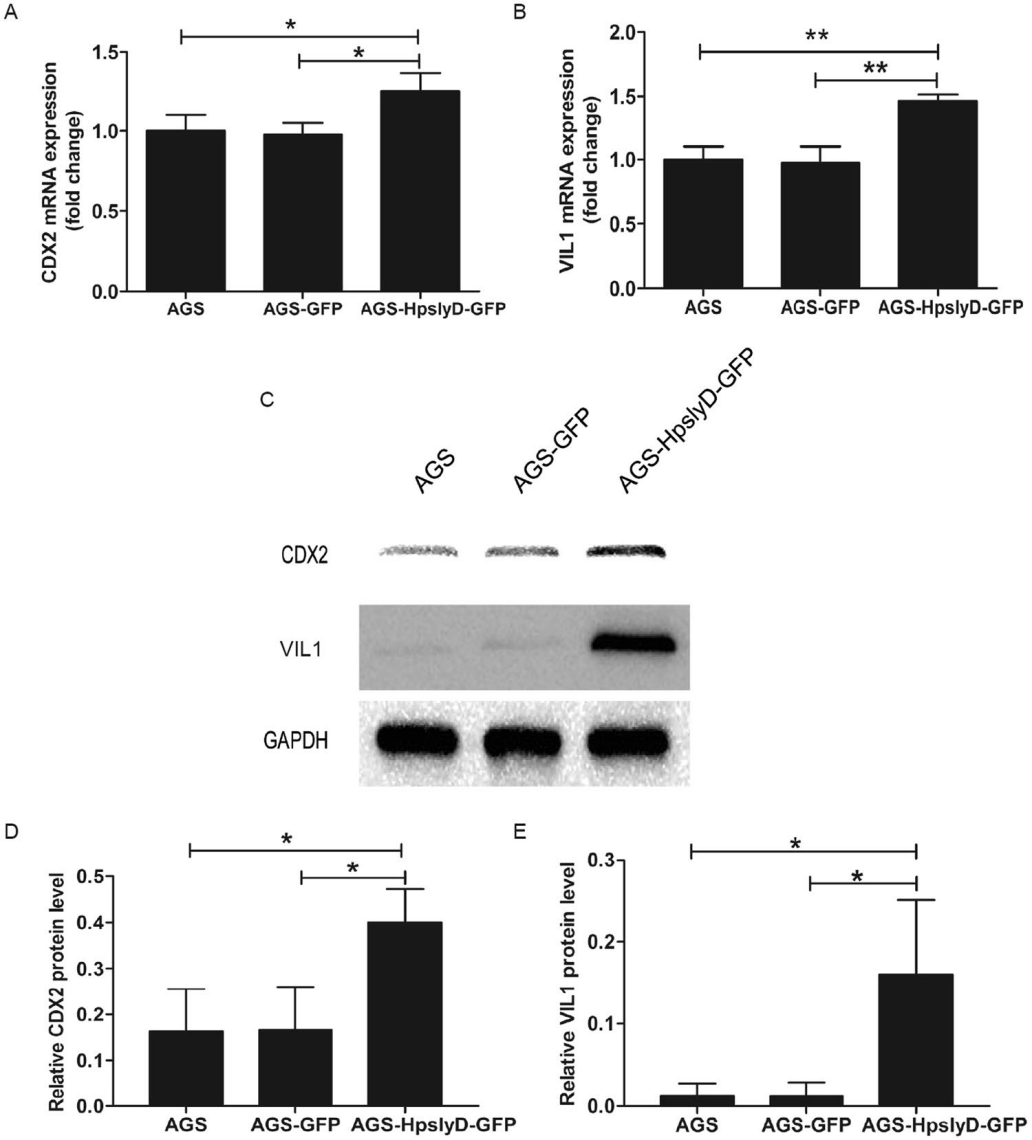
Expression of HpSlyD (stable cell lines) leads to increased expression of CDX2 and VIL1 in AGS cells. (**A,B**) Fold increase in *CDX2* (**A**) and *VIL1* (**B**) mRNA expression in AGS cells and AGS cells expressing either HpSlyD-GFP or GFP alone. The values obtained with the AGS cells are referred to as 1. *CDX2* and *VIL1* mRNA levels are normalized to *GAPDH* mRNA. (**C,D,E**) Western blots for CDX2 (**C,D**) and VIL1 (**C,E**) expression in AGS cells and AGS cells expressing either HpSlyD-GFP or GFP alone. AGS cells were used as a control and GAPDH was used as a loading control. The results shown in (**A,B,D,E**) are means ± SD, each experiment performed in triplicate. *P < 0.05, **P < 0.01 as compared with AGS cells.

The occurrence of gastric IM during *H. pylori* infection has been reported to be dependent on induction of CDX2 expression in gastric epithelial cells^30^. Thus, in initial studies, we evaluated CDX2 expression and the expression of another epithelial cell differentiation marker, VIL1, in human gastric cancer cell lines before and after treatment with HpSlyD. AGS or N87 cells were incubated with 200 µg/ml HpSlyD for 40 hours. The level of *CDX2* mRNA in the non-treated group was significantly lower than that of the treated group in both cell lines (Fig. 1A). Similarly, mRNAs encoding *VIL1* were up-regulated in the treated cells compared with the non-treated cells (Fig. 1B). In addition, CDX2 protein (as well as VIL1 protein) was also expressed at this time point (Fig. 1C–E). *CDX2* and *VIL1* mRNA expression in AGS cells expressing SlyD-GFP were significantly higher than in control AGS cells and AGS cells expressing GFP alone (Fig. 2A,B). The same differences were also found in the protein expression of CDX2 and VIL1 (Fig. 2C–E). Our results showed that in both gastric epithelial cell lines and HpSlyD stably expressing cell line, CDX2 and VIL1 expression was affected by the presence of HpSlyD.

### HpSlyD induced TCTP expression in human gastric epithelial cells

**Figure 3:**
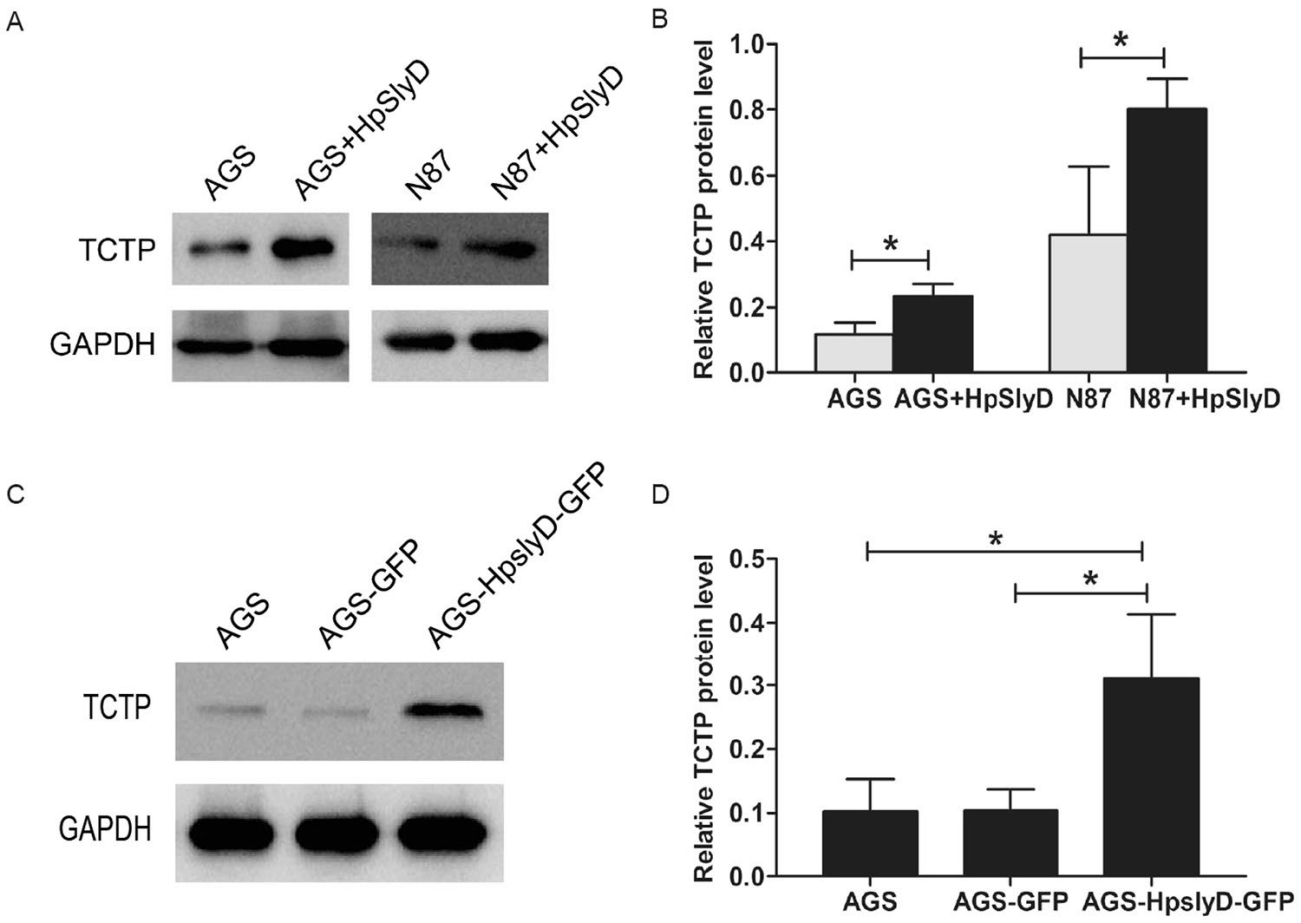
Effect of HpSlyD treatment on TCTP expression. (**A,B**) Western blot for TCTP expression in AGS and N87 cells treated with 200 µg/ml HpSlyD for 40 hours. Non-treated cells were used as a control. (**C**,**D**) Western blot for TCTP in AGS cells and AGS cells expressing either HpSlyD-GFP or GFP alone. AGS cells were used as a control. GAPDH was used as a loading control. Results shown in (**B,D**) are means ± SD, each experiment performed in triplicate. *P < 0.05, as compared with AGS cells. Full-length gels are presented in Supplemental Figure 2.

In our previous study, we found that TCTP is a highly expressed protein in an *HpslyD-GFP* stable cell line, suggesting that TCTP may be involved in *HpslyD*-mediated biological effects. With this information in hand, we next addressed whether HpslyD can induce increased TCTP expression in AGS, N87, and the *HpslyD-GFP* stable cell line. As shown in Fig. 3, TCTP expression was markedly increased in AGS and N87 cells treated with 200 µg/ml HpSlyD for 40 hours and in the *HpslyD-GFP* stable cell line, suggesting that HpSlyD affects TCTP expression in gastric epithelial cells.

### HpSlyD induction of CDX2 and VIL1 expression inhibited by knockdown of TCTP

**Figure 4:**
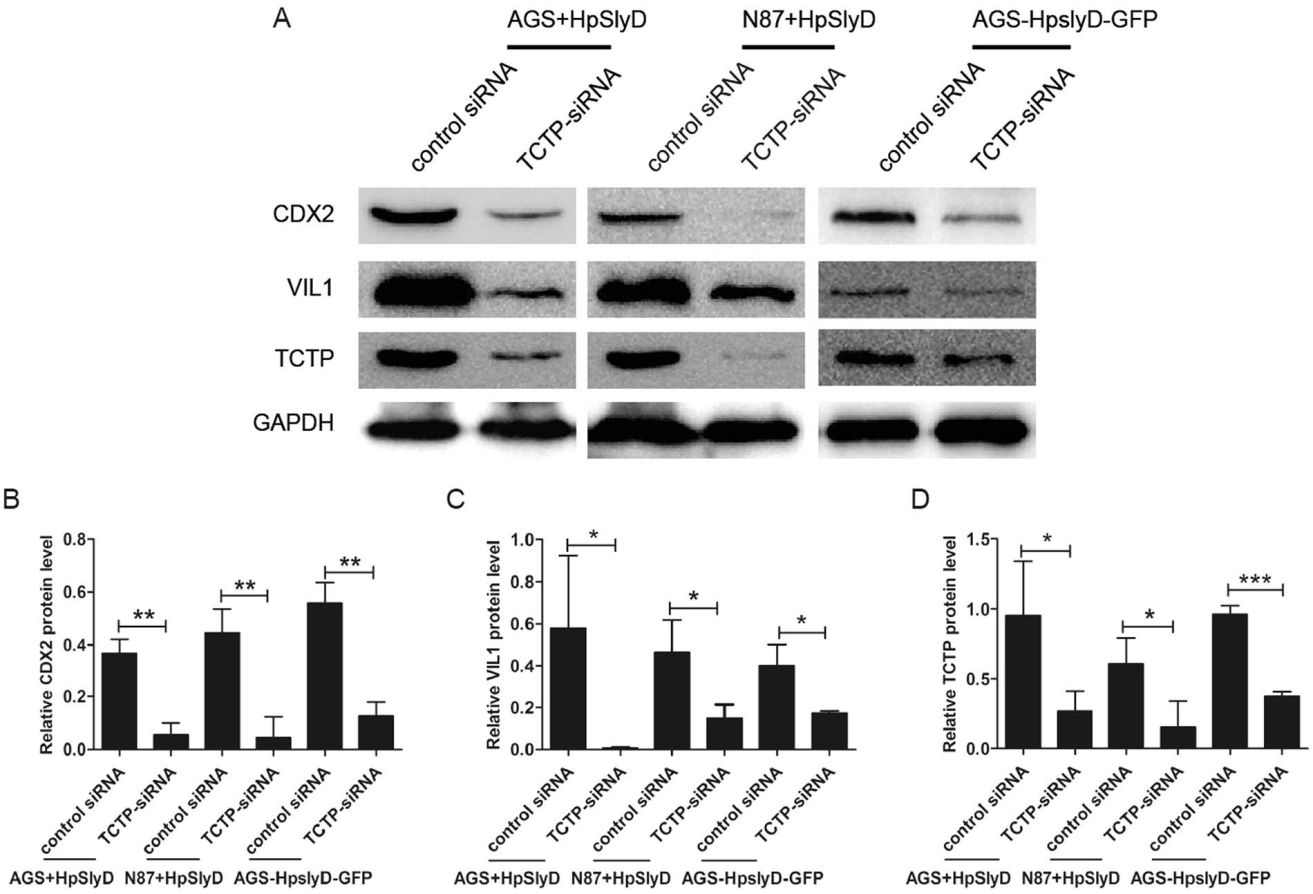
Knock down of TCTP blocks HpSlyD-induced expression of CDX2 and VIL1 in gastric epithelial cells. (**A**–**D**) Western blot for CDX2, VIL1 and TCTP in AGS, N87 and the stable *HpslyD-GFP* cell line after transfection with the indicated siRNA. GAPDH was used as a loading control. The results shown in B, C, D are the means ± SD, each experiment performed in triplicate. *P < 0.05, **P < 0.01, ***P < 0.001 as compared with control siRNA cells. Full-length gels are presented in Supplemental Figure 3.

To further examine whether TCTP regulates CDX2 and VIL1 expression induced by HpSlyD, we conducted a series of studies addressing the role of TCTP in HpSlyD induction of CDX2 and VIL1. AGS, N87, and AGS *HpslyD-GFP* stably expressing cell lines were transfected with TCTP siRNA or nonspecific siRNA for 6 hr and then treated with HpSlyD for another 40 hr. As shown in Fig. 4, TCTP-specific siRNA strongly inhibited HpSlyD-induced upregulation of CDX2 and VIL1, suggesting the involvement of TCTP in *H. pylori* induced CDX2 signalling. The same result can also be seen in both N87 cells and the *HpslyD-GFP* stably expressing cell line (Fig. 4A–D). Our data demonstrate that TCTP has a promotion effect on HpSlyD-induced CDX2 and VIL1 expression.

### TCTP introduction upregulated the expression of CDX2 and VIL1

**Figure 5:**
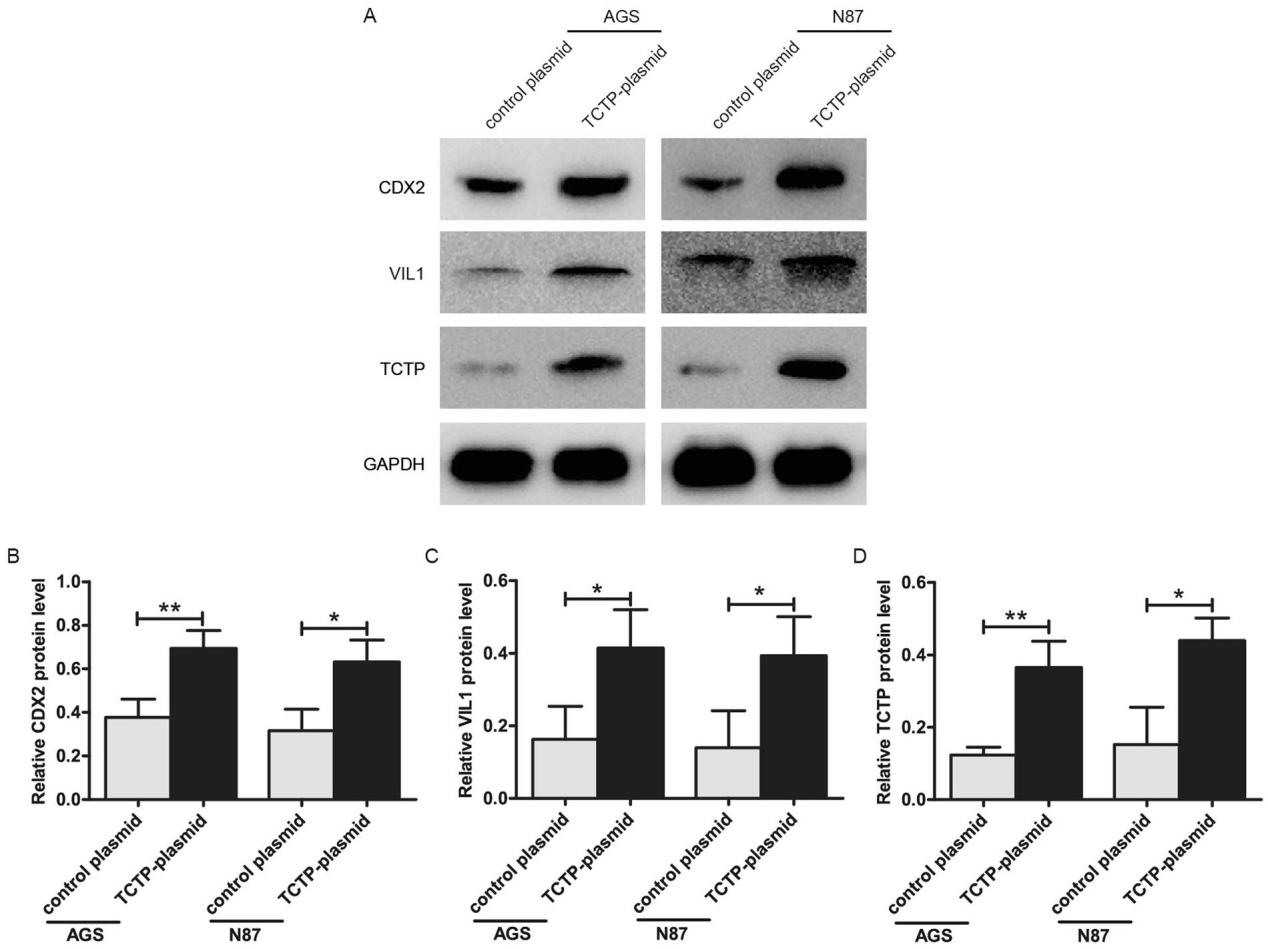
Effect of TCTP introduction on the expression of CDX2 and Villin. (**A**–**D**) Western blot for CDX2, VIL1 and TCTP in AGS, N87 after transfection with the indicated TCTP-plasmid or control plasmid. GAPDH was used as a loading control. The results shown in (**B,C,D**) are the means ± SD, each experiment performed in triplicate. *P < 0.05, **P < 0.01 as compared with control plasmid cells. Full-length gels are presented in Supplemental Figure 4.

The above results showed that TCTP was involved in HpSlyD-induced upregulation of CDX2 and VIL1. Whether the introduction of TCTP gene to the cell lines has the same biological effects as HpSlyD? We then transfected a TCTP expression plasmid and a control plasmid (Origene, China) into AGS and N87 cells using Lipofectamine 2000 (Invitrogen, USA). TCTP overexpresion was evaluated 24 hours after transfection by western blot. As shown in Fig. 5, TCTP introduction upregulated the CDX2 and VIL1 expression both in AGS and N87 cells, suggesting the involvement of TCTP in inducing CDX2 signaling. Our data demonstrate that TCTP overexpression has a promotion effect on CDX2 and VIL1 expression, just as the same biological effects as HpSlyD has.

### HpSlyD binding protein FK506 blocks HpSlyD-induced expression of CDX2, VIL1, and TCTP in AGS and N87 cells

**Figure 6:**
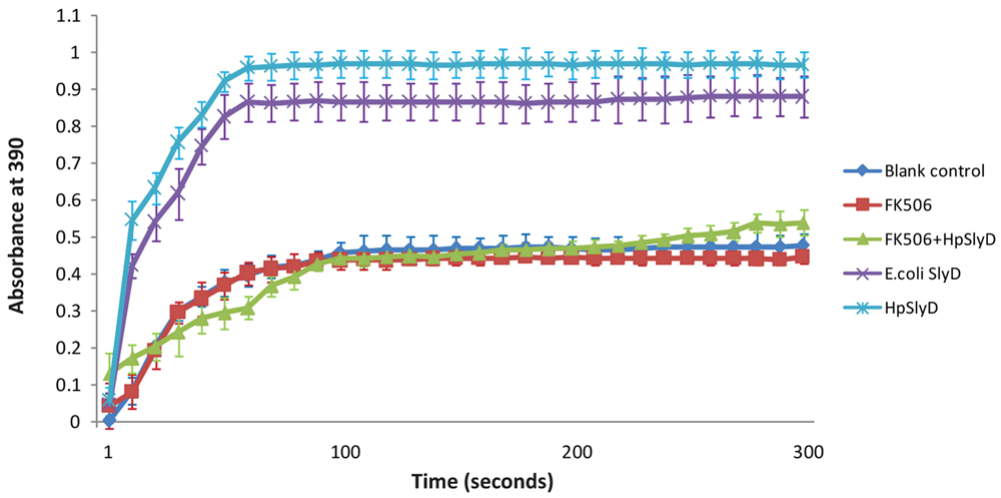
Tacrolimus (FK506) decreased the PPIase activity of HpSlyD. The activity of PPIase was measured as described in the Materials and Methods. Data are shown as the mean ± SD from three independent experiments. Note that a substantial decrease in PPIase activity is detected in the cells treated with HpSlyD + FK506.

FK506 can block the function of FK506-binding protein (FKBP) by binding to the immunophilin FKBP12^31–35^. HpSlyD is a member of the FKBP family. First, we assessed whether FK506 could inhibit HpSlyD enzymatic activity. As shown in Fig. 6, with *E. coli* SlyD as a positive control, enzymatic activity analysis revealed that PPIase activity was substantially lower in cells treated with HpSlyD+FK506 than in those treated with HpSlyD alone. Therefore, our data suggest that FK506 can suppress PPIase activation of HpSlyD.

**Figure 7:**
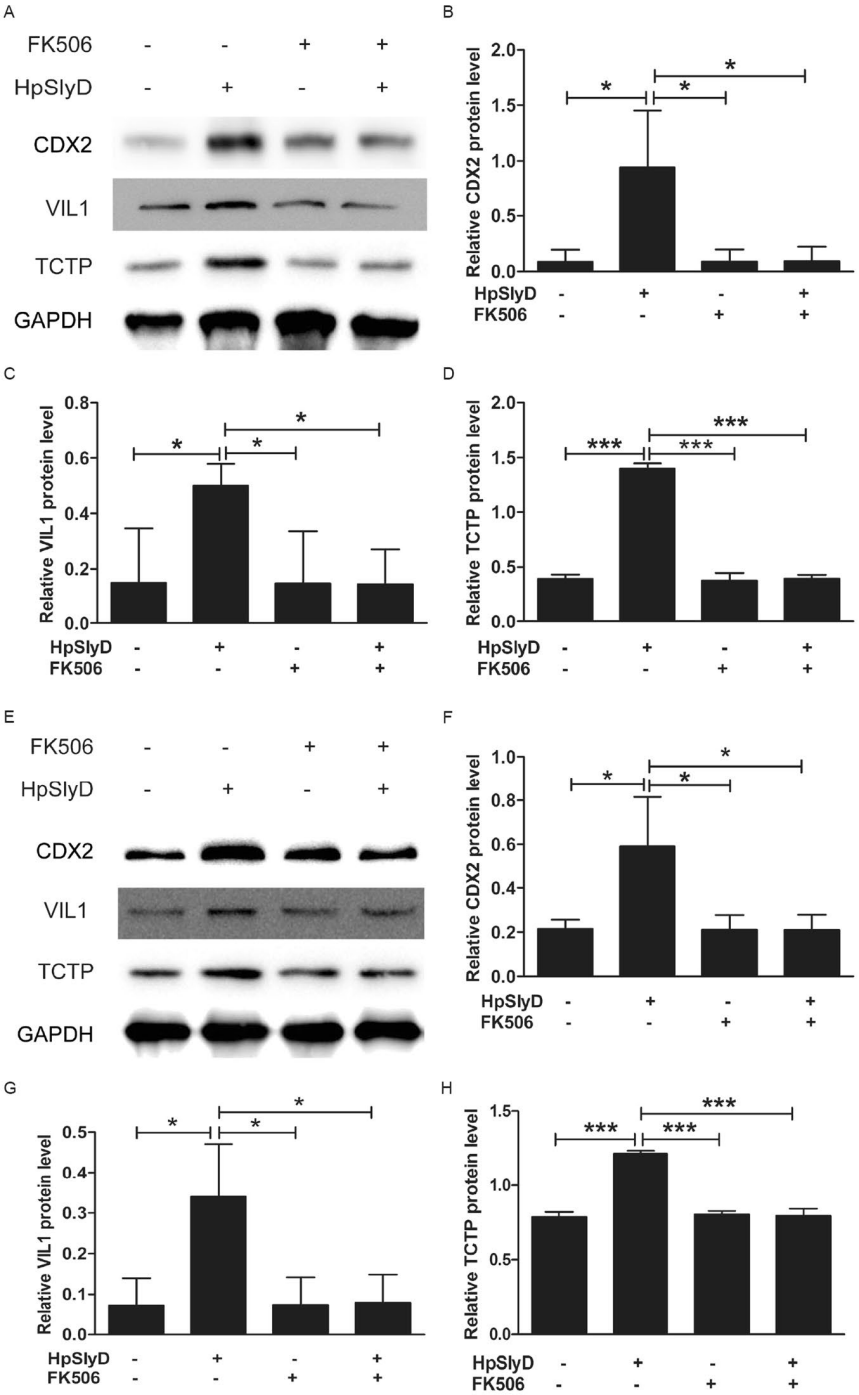
HpSlyD-induced expression of CDX2, VIL1 and TCTP in AGS and N87 cells is inhibited by HpSlyD binding protein, FK506. Western blot for CDX2, VIL1 and TCTP expression in AGS (**A**–**D**) or N87 (**E**–**H**) cells treated with either 200 µg/ml HpSlyD or FK506 or both. Non-treated cells were used as a control and GAPDH was used as a loading control. The results shown in (**B**–**D,F**–**H**) are means ± SD, each experiment performed in triplicate. *P < 0.05, ***P < 0.001 as compared with non-treated cells.

We next addressed the effect of FK506 on HpSlyD-induced expression of CDX2, VIL1 and TCTP. As shown in Fig. 7, co-treatment of cells with HpSlyD and FK506 led to significant reductions in CDX2, VIL1 and TCTP expression compared with cells treated with HpSlyD alone in both the AGS (Fig. 7A–D) and N87 (Fig. 7E–H) cell lines. Thus, FK506 as a binding protein of HpSlyD does block HpSlyD-induced expression of CDX2, VIL1 and TCTP.

### HpSlyD related to the expression of CDX2 and TCTP in different gastric diseases

The above *in vitro* studies showed that HpSlyD induces CDX2 and VIL1 expression mediated through TCTP. To determine if a similar phenomenon occurs *in vivo* we immunostained human different gastric diseases tissue with or without HpSlyD infection. The information from the patients’ included in this study is summarized in Supplement Table 1. There was no statistically significant difference in age and sex between groups.

**Figure 8:**
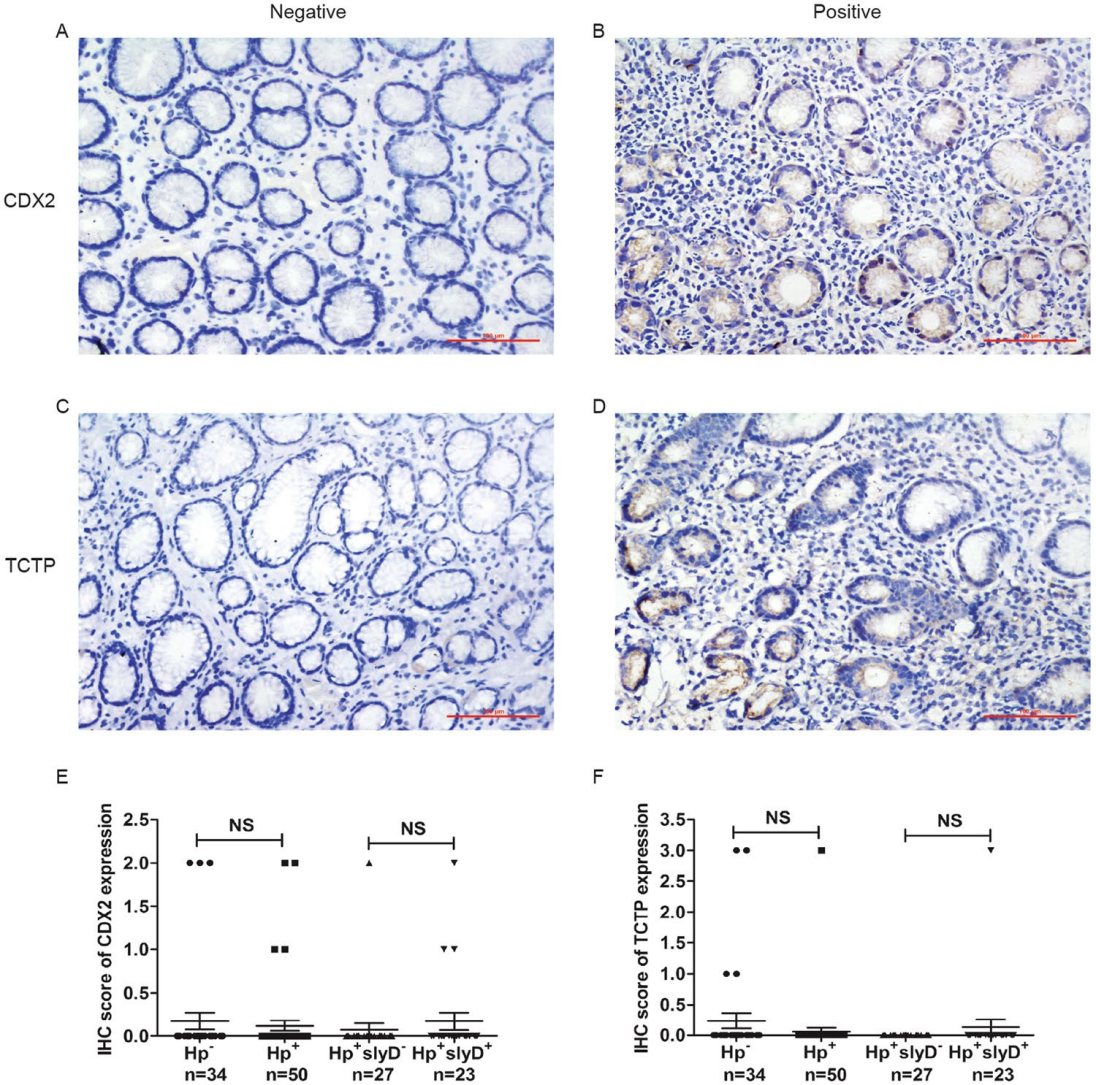
IHC staining of CDX2 and TCTP in the GS of gastric mucosa. CDX2 was detected in the nucleus; however, TCTP was detected primarily in the cytoplasm (magnification: ×200). (**A,B**) Representative photomicrographs of IHC staining of CDX2. (**C,D**) Representative photomicrographs of IHC staining of TCTP. (**E**) Boxplot shows that CDX2 expression is no statistical difference in *H. pylori* positive and HpslyD positive cases than in negative ones. (**F**) Boxplot shows that TCTP expression is no statistical difference in *H. pylori* positive and HpslyD positive cases than in negative ones. NS, no statistical significance.

In GS group, the IS of CDX2 expression was no statistical difference no matter in *H*. *pylori* positive cases than in the negative ones or in the *HpslyD* positive cases than in the negative ones (P > 0.05, Fig. 8A–E). The IS of TCTP expression was also no difference between *H. pylori* groups (P > 0.05, Fig. 8C,D,F). These results indicated that the HpslyD positive *H. pylori* strain doesn’t promotes the expression of CDX2 and TCTP in GS.

**Figure 9:**
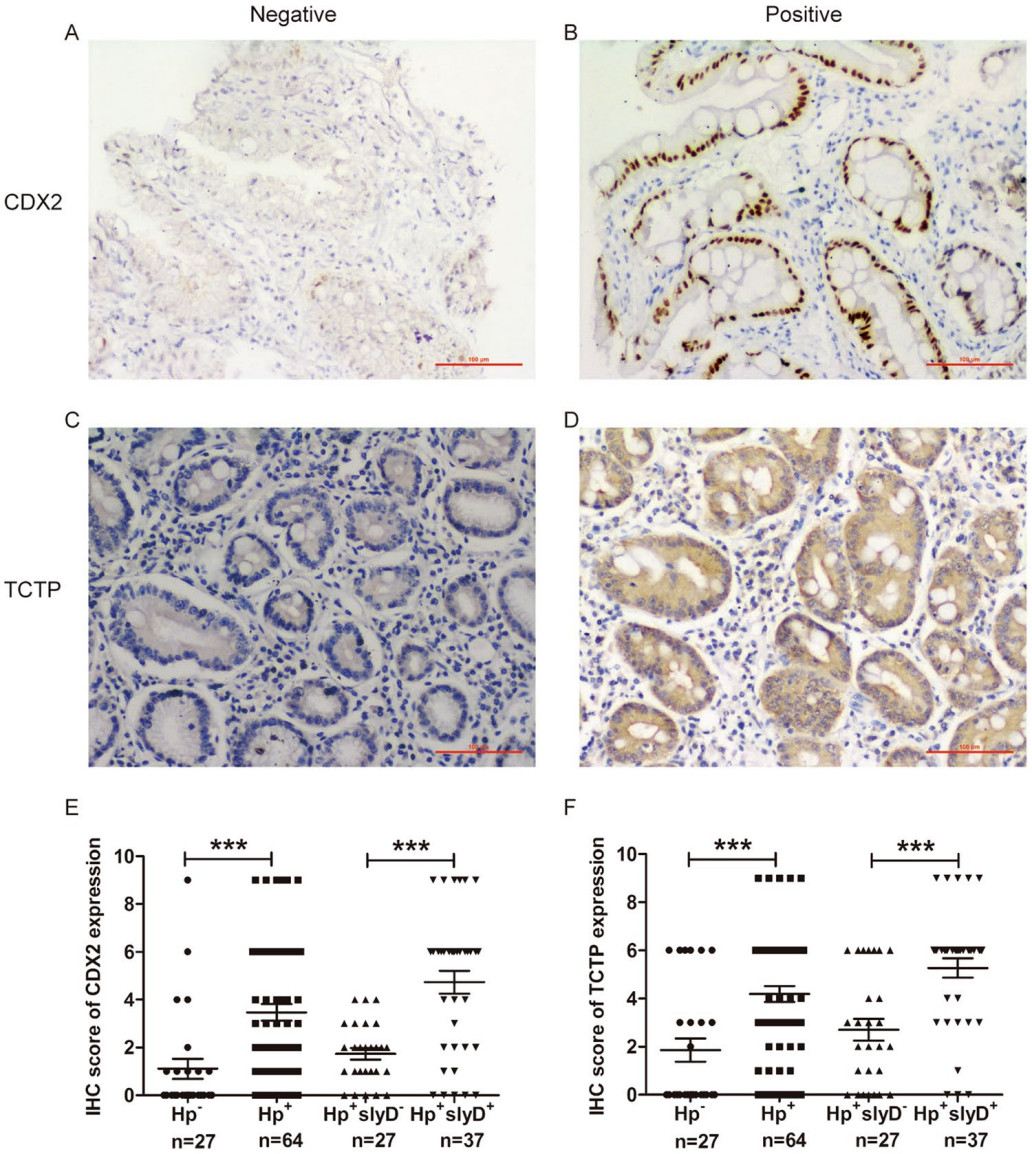
IHC staining of CDX2 and TCTP in the IM of gastric mucosa. (**A,B**) Representative photomicrographs of IHC staining of CDX2. (**C,D**) Representative photomicrographs of IHC staining of TCTP. (**E**) Boxplot shows that CDX2 expression is significantly higher in *H. pylori* positive and *HpslyD* positive cases than in negative ones. (**F**) Boxplot shows that TCTP expression is significantly higher in *H. pylori* positive and *HpslyD* positive cases than in negative ones. ***P < 0.001.

In IM-GA group, the IS of CDX2 expression was higher not only in *H. pylori* positive cases than in the negative cases but also in the *HpslyD* positive cases than in the negative group (P < 0.001, Fig. 9A,B,E). The same expression trend can also be seen in the IS of TCTP expression (P < 0.001, Fig. 9C,D,F). These results show that *HpslyD* positive *H. pylori* strain promotes the expressions of CDX2 and TCTP in IM-GA.

**Figure 10:**
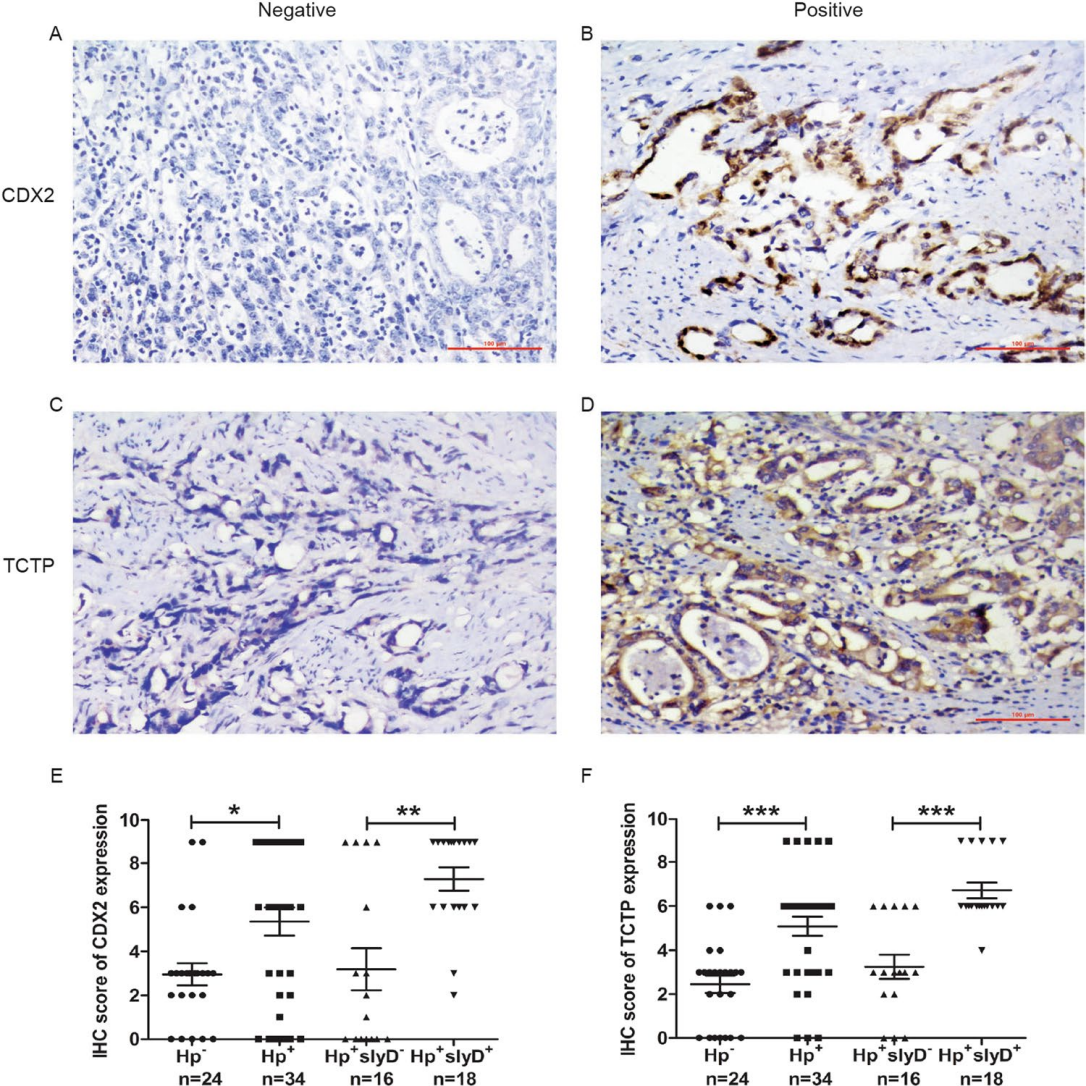
IHC staining of CDX2 and TCTP in the GC of gastric mucosa. (**A,B**) Representative photomicrographs of IHC staining of CDX2. (**C,D**) Representative photomicrographs of IHC staining of TCTP. (**E**) Boxplot shows that CDX2 expression is significantly higher in *H. pylori* positive and *HpslyD* positive cases than in negative ones. (**F**) Boxplot shows that TCTP expression is significantly higher in *H. pylori* positive and *HpslyD* positive cases than in negative ones. *P < 0.05, **P < 0.01, ***P < 0.001.

In GC group, the IS of CDX2 expression was higher in *H. pylori* positive specimens than in the negative specimens, and higher in *HpslyD* positive specimens than in the negative specimens (P < 0.05 and P < 0.01, Fig. 10A,B,E). The same expression trend can also be seen in the IS of TCTP expression (P < 0.001, Fig. 10C,D,F). These results show that HpslyD positive *H. pylori* strain promotes the expressions of CDX2 and TCTP in GC.

**Figure 11:**
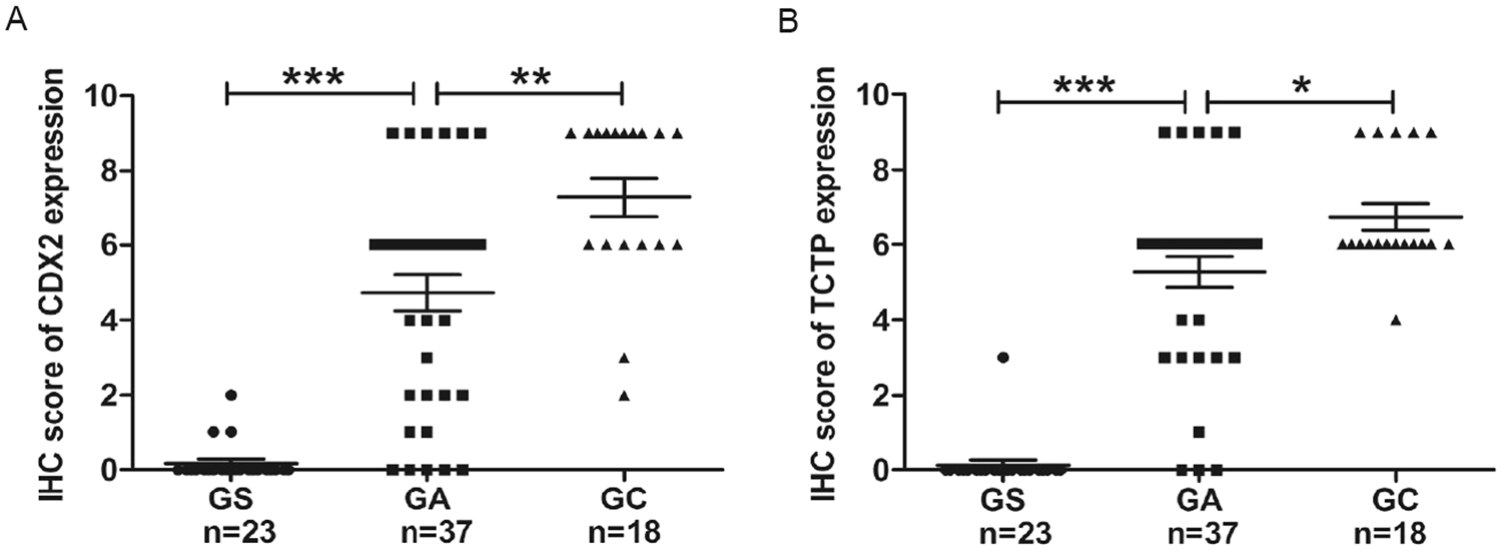
CDX2 and TCTP expression of different gastric diseases in HpslyD positive. (**A**) Boxplot shows that CDX2 expression of HpslyD positive is significantly higher in GC than that in IM-GA, and also is significantly higher in IM-GA than that in GS. (**B**) Boxplot shows that TCTP expression of HpslyD positive is significantly higher in GC than that in IM-GA, and also is significantly higher in IM-GA than that in GS. *P < 0.05, **P < 0.01, ***P < 0.001.

And then we compared TCTP and CDX2 expressions of different gastric diseases in *HpslyD* positive. As shown in Fig. 11, the IS of CDX2 and TCTP expressions are significantly higher in GC than that of IM-GA, which is also significantly higher in IM-GA than that of GS, indicating that the HpslyD positive *H. pylori* strain promotes the expression of CDX2 and TCTP with the development of gastric diseases.

### TCTP is positively correlated with CDX2 in *H. pylori slyD* positive infection

**Figure 12:**
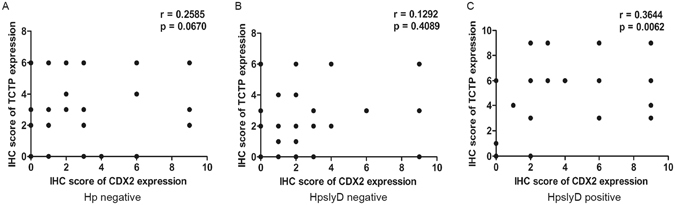
A scatter plot of TCTP and CDX2 expression. The scatter plot shows a positive correlation between TCTP and CDX2 levels in cases positive for *HpslyD* infection.

We next evaluated the relationship between TCTP and CDX2 expression. As shown in Fig. 12, we identified a positive correlation between TCTP and CDX2 levels in *HpslyD* positive cases (Spearman’s correlation coefficient, r = 0.3644, P < 0.01) but not in *HpslyD* negative cases (r = 0.1292, P = 0.4089) or *H. pylori* negative cases (r = 0.2585, P = 0.067).

## Discussion

In a previous study, we identified *HpslyD* as a gastric cancer-associated gene^6^. Further study has shown that infection with *slyD*-positive *H. pylori* strains is associated with atrophic gastritis^9^. However, the mechanism by which HpslyD provokes metaplastic changes is poorly understood. In this study, we fill this gap with studies showing that HpSlyD induces CDX2 and VIL1 expression both *in vitro* and *in vivo*. In addition, this study is the first to confirm that the TCTP-mediated signalling pathway is involved in HpSlyD-induced IM in the stomach. These results provide novel information that contributes to understanding the molecular events that precede the development of gastric diseases caused by *H. pylori* infection.

Metaplasia is a process whereby a completely differentiated cell transforms into another type of mature cell, and this process is stimulated by certain factors in response to environmental changes. IM refers to a series of phenotype changes from stomach epithelium to an intestinal phenotype during the process of changing from gastritis to atrophic gastritis and sometimes to intestinal-type gastric cancer. This change is caused by an integration of genetic factors expression, transcription factors, signalling pathways and growth factors. CDX2 is a homeobox transcription factor that is critical for intestinal differentiation^36, 37^, and is a specific biomarker of the early steps of the gastric carcinogenic cascade, driving the development of IM^38, 39^. The key role of CDX2 in the metaplastic transformation of the gastric mucosa was categorically demonstrated by the use of two transgenic mouse models with ectopic expression of CDX2 in the gastric epithelium and subsequent development of IM with absorptive, goblet and enteroendocrine cell types^40, 41^. VIL1 is a structural protein involved in the formation of small intestinal microvilli and its expression is upregulated in IM. VIL1 is a known transcriptional target of CDX2^11^. Using two kinds of gastric epithelial cells *in vitro* we showed that HpSlyD induced CDX2 and VIL1 expression. Furthermore, a similar result was confirmed in an *HpslyD* stable cell line, which we constructed in previous studies. Therefore, our results indicate that the expression of CDX2 and VIL1 is associated with the presence of HpSlyD.

SlyD, as a multifaceted protein, belongs to the PPIase FKBP family and catalyses the intrinsically slow *cis*–*trans* isomerization of peptidylprolyl bonds (Xaa-Pro) to facilitate the protein folding process^42, 43^, but its role as a PPIase *in vivo* is not well understood. Previous functional and interactional studies have shown that HpSlyD is involved in nickel ion integration of urease and hydrogenase^42, 44^. Our previous studies showed that *HpslyD* is a high-copy gene in gastric cancer patients by constructing a gastric cancer-related *H. pylori* differential gene library and that HpSlyD influences the gastric cell biological processes of cell proliferation, transformation and migration^7, 8^. Recently, some researchers demonstrated an emerging role of mammalian PPIase in cell differentiation^45^ and therefore bacterial-derived PPIase may also be involved in phenotype transitions. The present study suggests that HpSlyD regulates CDX2 and VIL1 to promote IM transition in gastric epithelial cells. This study broadens our understanding of bacterial-derived PPIase and provides a theoretical basis for understanding the function of HpSlyD and an in-depth exploration of the pathogenesis of *H. pylori*.

The molecular mechanism of *H*. *pylori*’s regulation of CDX2 expression has been reported in the literature. Camilo *et al*. have demonstrated that *H. pylori* infection and the role of the BMP pathway in the regulation of intestinal and gastric-specific genes that might be relevant for gastric IM^30^. Asano *et al*. have reported that *H*. *pylori* infection induces CDX2 expression and intestinal metaplasia of gastric cells by NOD1-mediated innate immune responses^13^. Rieder *et al*. found *H. pylori* induction of VIL1 in the stomach correlates with the activation and cooperative binding of ELK1 and SRF to the proximal promoter of VIL1 during the process of developing intestinal metaplasia^12^. Thus, CDX2 and VIL1 expression regulated by *H. pylori* is a relatively complex process involving the interaction of many signalling pathways. However, the signalling pathway involved in HpSlyD-induced CDX2 and VIL1 expression is not yet completely understood. TCTP is at the heart of the cell-reprogramming network, playing the role of a checkpoint, and is involved in regulating transition points of cell phenotypes under a variety of physiological or pathological states. *In vitro*, we found that TCTP expression was markedly increased in AGS and N87 cells treated with HpSlyD and in an *HpslyD* stable cell line, suggesting that HpSlyD also affects TCTP expression in gastric epithelial cells. Meanwhile, we observed that downregulation of TCTP protein led to decreased HpSlyD-induced CDX2 and VIL1 expression and overexpression of TCTP improved the levels of CDX2 and Villin. Co-treatment with HpSlyD and FK506 led to a significant reduction in CDX2, VIL1 and TCTP expression. Furthermore, IHC staining demonstrated that CDX2 and TCTP expression were higher in *H. pylori* positive specimens than in *H. pylori* negative specimens, and higher in *HpslyD* positive specimens than in *HpslyD* negative specimens. *HpslyD* positive *H. pylori* strain promotes the expression of CDX2 and TCTP with the development of gastric diseases. In *HpslyD* positive specimens, the expression of CDX2 was positively correlated with TCTP. Our results show that HpSlyD induces CDX2 and VIL1 expression mediated through TCTP and contributes to IM and the development of gastric diseases. We can further speculate that HpSlyD can activate cell differentiation mediated by transcriptional factors through TCTP, re-programming gastric epithelial cells from the gastric phenotype to the intestinal phenotype. This process may also be involved in the malignant transformation of gastric tissue harbouring this chronic and stable infection.

In conclusion, we demonstrated that *H. pylori* infection leads to increased expression of CDX2 and VIL1 and that TCTP enhances this expression, and these changes were associated with the development of IM and cancer in the gastric mucosa. The results presented in this study show that HpSlyD is a positive regulator of IM progression, and therefore, it may be a possible therapeutic target for inhibiting the formation of IM after *H. pylori* infection. Our results provide novel information for understanding the molecular events that precede the development of gastric IM, reinforcing the role of the HpSlyD-TCTP-CDX2 pathway in the whole process. Our study also provides an important molecular target for the clinical monitoring of *H. pylori* infection and ‘type-based therapy’, and provides insight into ideas and strategies for blocking *H. pylori*-related IM formation and decreasing the risk of progression to gastric cancer.

## Electronic supplementary material

Supplemental information
