# Supplementary material for: HpSlyD inducing CDX2 and VIL1 expression mediated through TCTP protein may contribute to intestinal metaplasia in the stomach
Source: Sci Rep. 2017 May 23;7:2278. doi: 10.1038/s41598-017-02642-y (PMC5442128; doi:10.1038/s41598-017-02642-y)
Supplement: Supplementary file 1 — Supplemental information [file 41598_2017_2642_MOESM1_ESM.doc]

**HpSlyD inducing CDX2 and VIL1 expression mediated through TCTP protein may contribute to intestinal metaplasia in the stomach**

**Running title: HpSlyD inducing CDX2, VIL1, and TCTP contribute to IM**

Qiuping Li^1^, Yanmei Zhu^1,3^, Jun Liu^2^, Xiuwen Yu^1, 4^, Moye Chen^1^, Nannan Dong^1^, Yuehua Gong^1, *^, Yuan Yuan^1, *^

^1^Tumor Etiology and Screening Department of Cancer Institute and General Surgery, the First Affiliated Hospital of China Medical University, and Key Laboratory of Cancer Etiology and Prevention (China Medical University), Liaoning Provincial Education Department, Shenyang 110001, China

^2^Mary Babb Randolph Cancer Center, West Virginia University, Morgantown, WV 26506, USA and Department of Physiology and Pharmacology, West Virginia University, Morgantown, WV 26506-9229, USA

^3^Department of Pathology, Cancer Hospital of China Medical University; Liaoning Cancer Hospital & Institute, Shenyang, 110042, Liaoning Province, China

^4^Department of Pathology, Qiqihar Medical College, Qiqihar, Heilongjiang, China

*Co-Correspondence should be addressed to

Yuehua Gong: yhgong@cmu.edu.cn

Yuan Yuan：[yuanyuan@cmu.edu](mailto:yuanyuan@cmu.edu).cn

| **Supplemental Table 1. General information of different patient groups** | | | | | | |
| --- | --- | --- | --- | --- | --- | --- |
| **Group** | **Characteristic** | **Total** | ***Hp* negative(%)** | ***HpslyD* negative(%)** | ***HpslyD* positive(%)** | **P value** |
| GS | Age |  |  |  |  | 0.74 |
|  | ≤60 | 60 | 25(41.7) | 20(33.3) | 15(25.0) |  |
|  | ＞60 | 24 | 9(37.5) | 7(29.2) | 8(33.3) |  |
|  | Gender |  |  |  |  | 0.134 |
|  | Female | 41 | 16(39.0) | 17(41.5) | 8(19.5) |  |
|  | Male | 43 | 18(41.9) | 10(23.3) | 15(34.9) |  |
|  | Total | 84 | 34(40.5) | 27(32.1) | 23(27.4) |  |
| IM-GA | Age |  |  |  |  | 0.308 |
|  | ≤60 | 62 | 19(30.6) | 21(33.9) | 22(35.5) |  |
|  | ＞60 | 29 | 8(27.6) | 6(20.7) | 15(51.7) |  |
|  | Gender |  |  |  |  | 0.304 |
|  | Female | 38 | 12(31.6) | 8(21.1) | 18(47.4) |  |
|  | Male | 53 | 15(28.3) | 19(35.8) | 19(35.8) |  |
|  | Total | 91 | 27(29.7) | 27(29.7) | 37(40.7) |  |
| GC | Age |  |  |  |  | 0.265 |
|  | ≤60 | 27 | 14(51.9) | 7(25.9) | 6(22.2) |  |
|  | ＞60 | 31 | 10(32.3) | 9(29.0) | 12(38.7) |  |
|  | Gender |  |  |  |  | 0.247 |
|  | Female | 21 | 6(28.6) | 6(28.6) | 9(42.9) |  |
|  | Male | 37 | 18(48.6) | 10(27.0) | 9(24.3) |  |
|  | Total | 58 | 24(41.4) | 16(27.6) | 18(31.0) |  |

**Supplemental Figure**

**
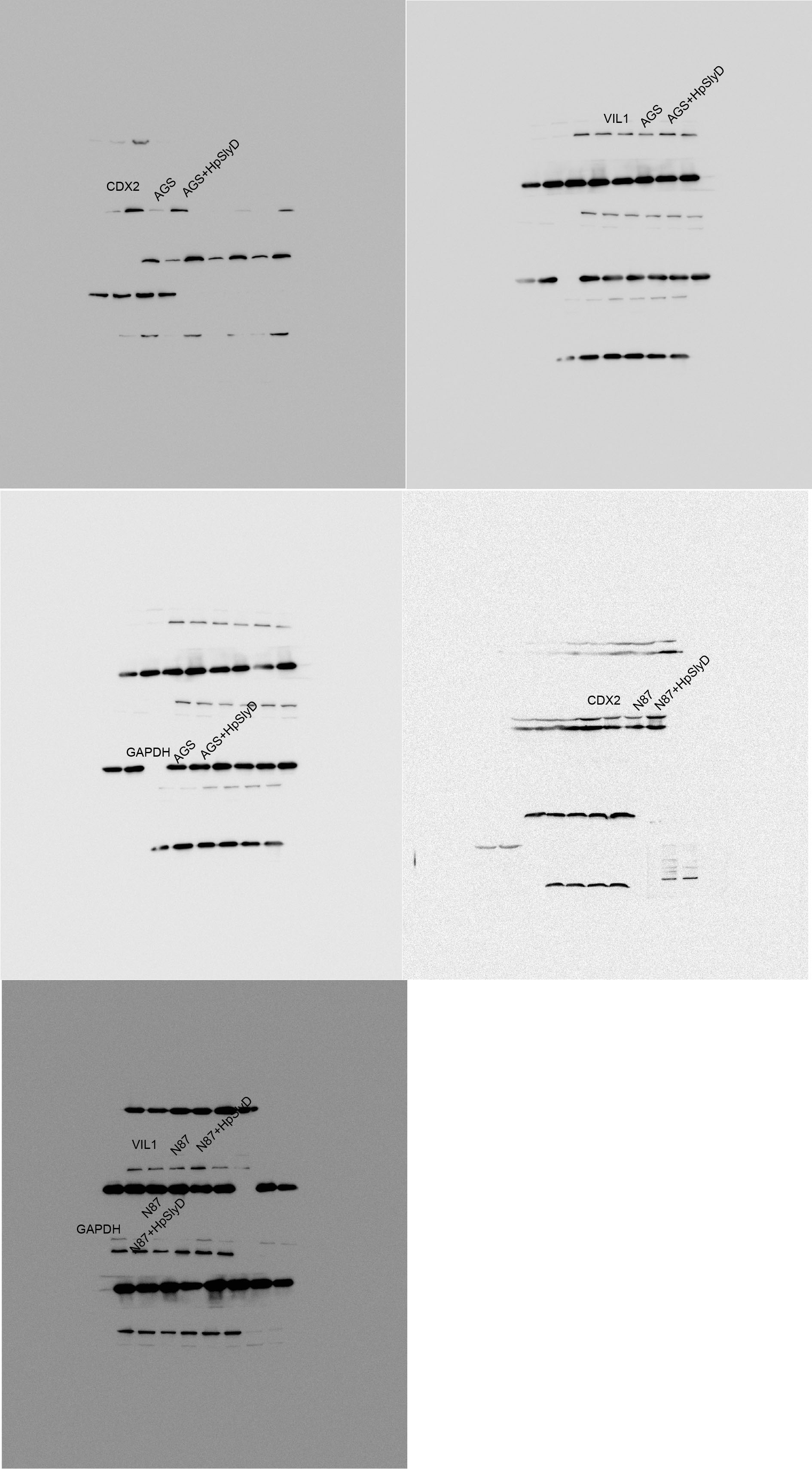
**

Supplemental Figure 1. Full-length gels of Figure 1C.


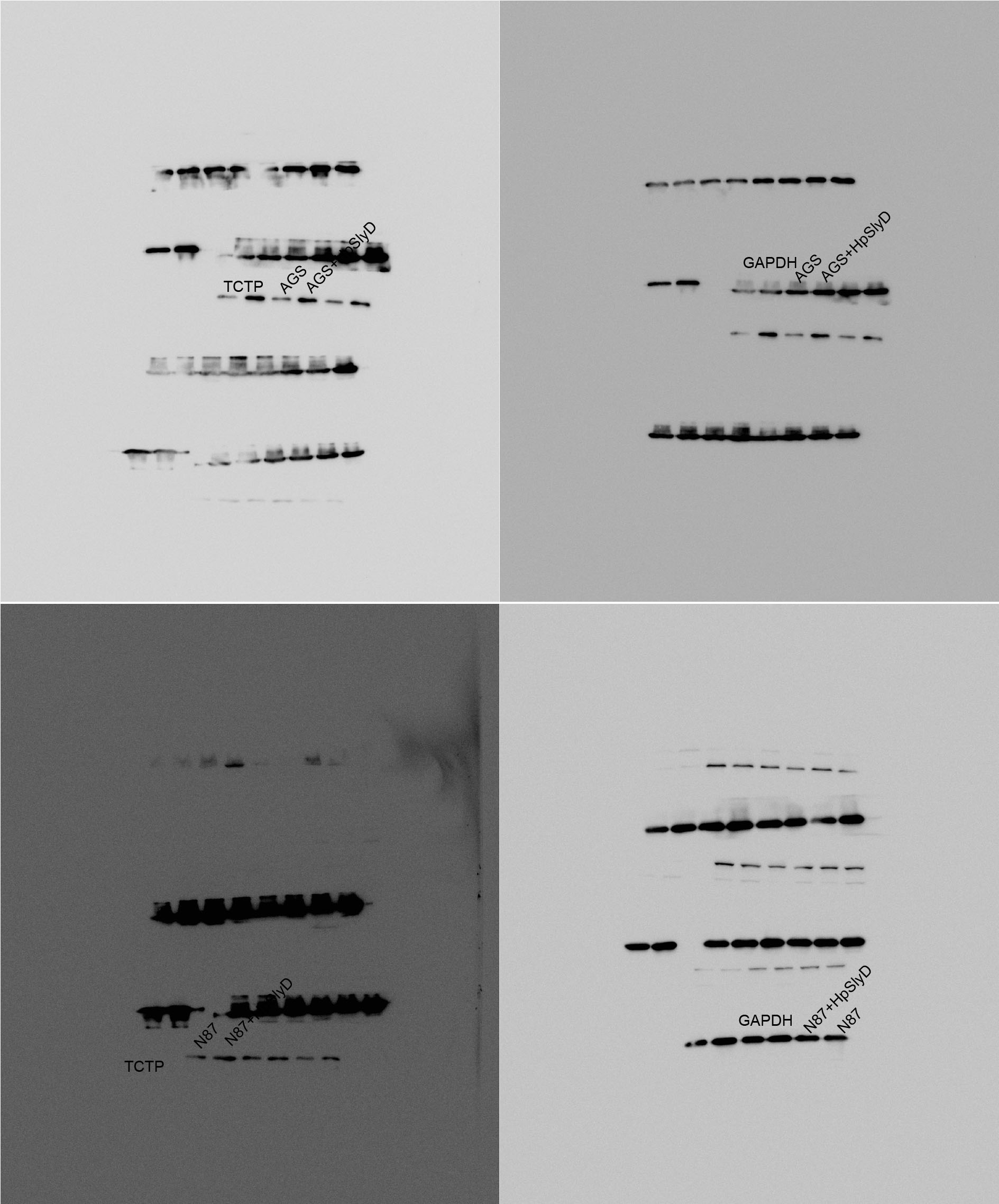


Supplemental Figure 2. Full-length gels of Figure 3A.


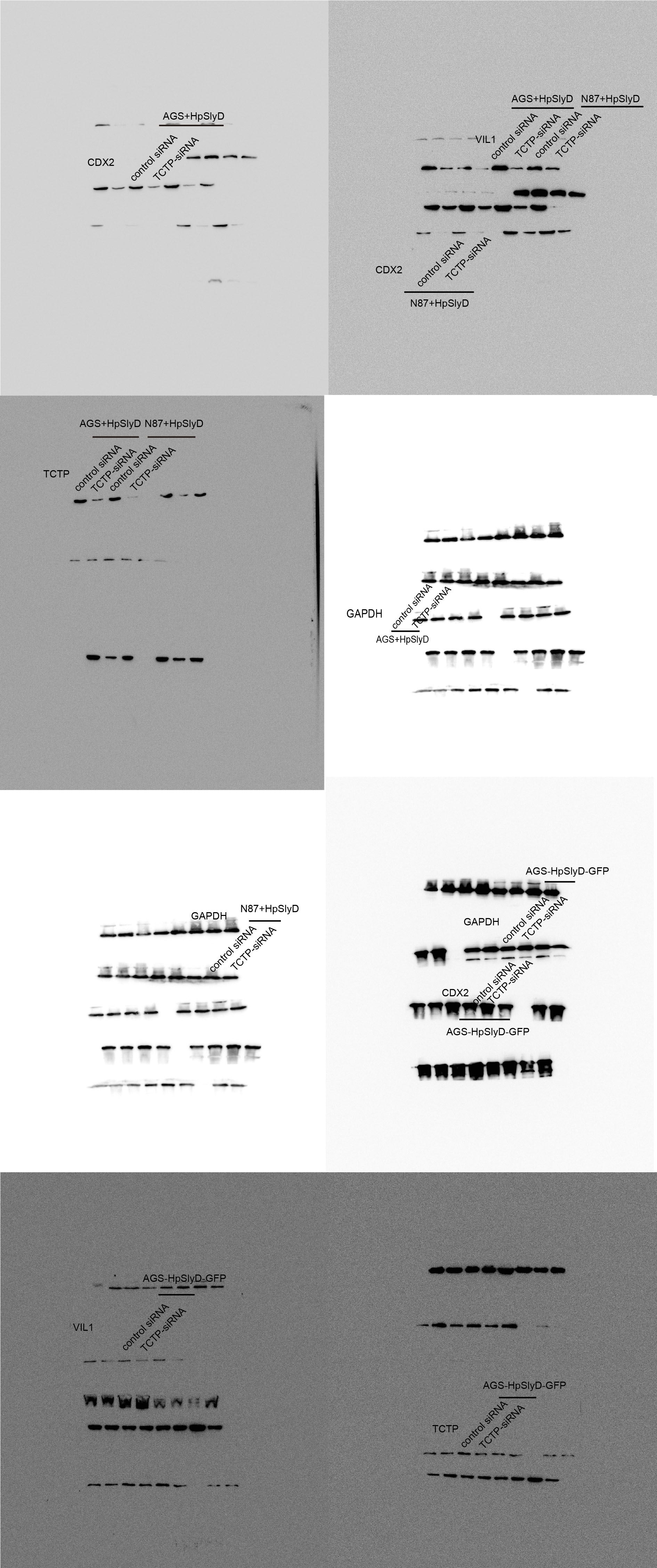


Supplemental Figure 3. Full-length gels of Figure 4A.


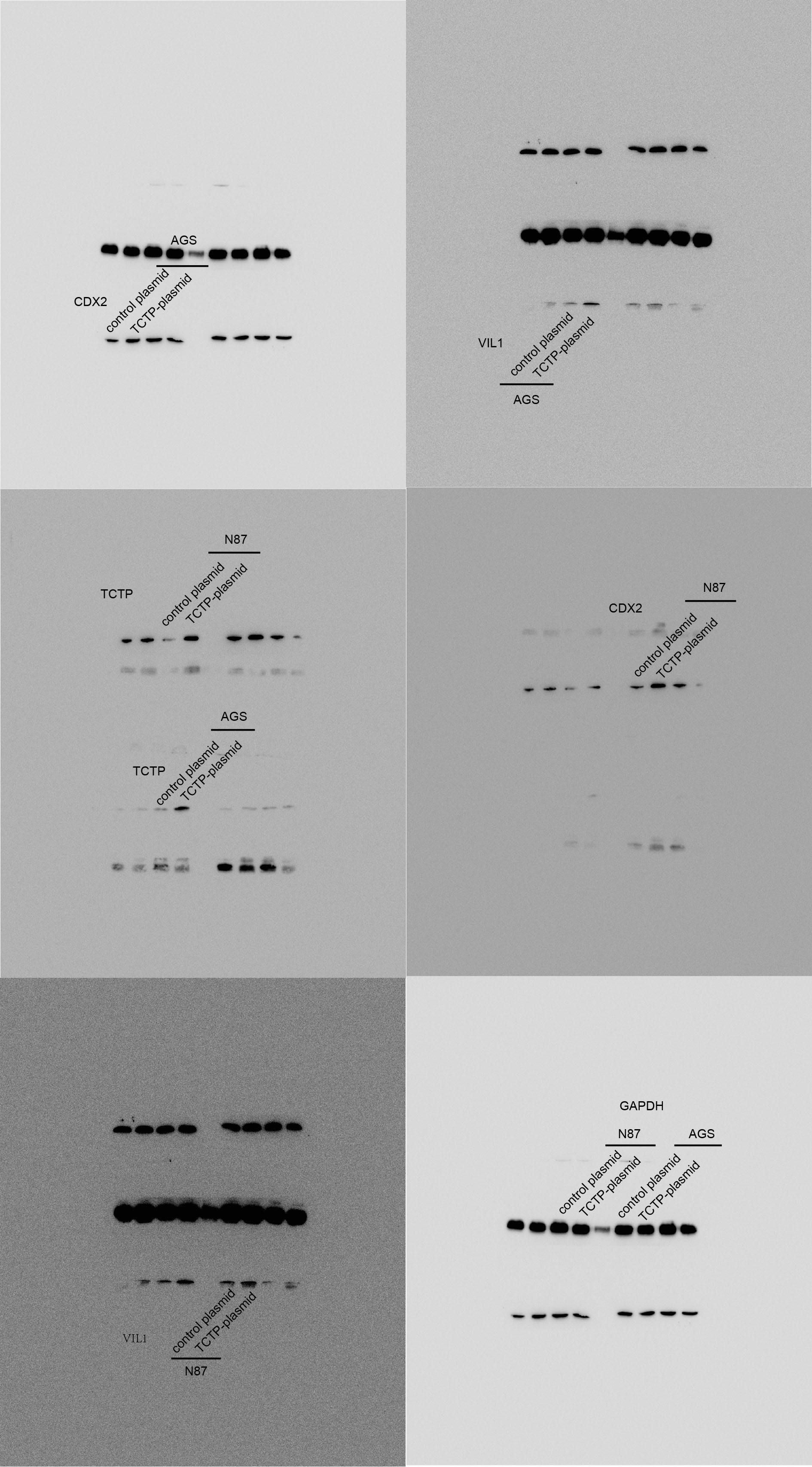


Supplemental Figure 4. Full-length gels of Figure 5A.
